# Methods, methodological challenges and lesson learned from phenomenological study about OSCE experience: Overview of paradigm-driven qualitative approach in medical education

**DOI:** 10.1016/j.amsu.2019.11.013

**Published:** 2019-11-23

**Authors:** Getu Ataro

**Affiliations:** Hawassa University College of Medicine and Health Sciences, Department of Anesthesia, Hawassa, Ethiopia

**Keywords:** Research paradigms, Research approaches, Medical education research, Descriptive phenomenology, Collaizi's descriptive phenomenological analysis

## Abstract

Qualitative research approach could be as important as quantitative one, particularly in medical education, as long as it meets the common goal of both—improving the quality of education. In contrary to the end—i.e. achieving the common goals, the means of both approaches of inquiry is different. Their dissimilarity in the means or process is not confined to data collection techniques, study designs or analysis methods; but, they also differ in assumptions about the world, reality, science and knowledge. Implicitly or explicitly, these assumptions are revealed in a researcher's discussion about philosophical assumptions and research paradigms. The researcher's inclination towards any of paradigms and assumption in light of the most common philosophical concepts such as ontology, epistemology and methodology results in choice of either of the dominant research paradigms to follow such as objectivism/positivism and interpretivisim/constructivism. This is common practice in the quantitative-qualitative dichotomy of research world disregarding the emerging mixed approach with predominantly pragmatism paradigm. Besides framing the methodology of the study, researcher's explicit description of philosophical assumptions and paradigms helps readers easily understand study findings. Many authors from both dominant traditions fail to describe this important aspect of the research in their published works. In our study, the ontological and epistemological assumptions led us choose interpretivist/constructivist paradigm and phenomenological qualitative approach with Collaizi's descriptive phenomenological analysis adapted to our context. The experience and lesson learned from the study found to be worse sharing in a modified and extended construct of methodology part. Therefore, this article deals with philosophical positions, research paradigms and traditions that led to the specific qualitative approach from the perspective of methodology part in our study about objective structure clinical examination (OSCE) experience in a medical department.

## Highlights

• Qualitative research approach can be as important as quantitative approach.
• A research approach can be determined by preference to certain research paradigm.
• Major philosophical concepts in research are ontology, epistemology and methodology.
• Disclosing one's research paradigm help research readers easily understand findings.
• Collaizi's seven step descriptive phenomenology fits for our OSCE experience study.

## 1 Introduction

Qualitative research approach is overwhelmingly dominated by positivist-driven quantitative approach in studies published on medical education journals. Evidence shows, however, there is no consensuses among scholars whether or not qualitative approach is superior to the quantitative one and vice versa as far as science education (including health professions education) research is concerned, instead, both have different assumptions and strategies ultimately leading to the achievement of the same goal—improved quality of education [1]. On the other hand, unlike it has been perceived by many, difference in qualitative and quantitative methods is beyond the difference in data collection techniques (e.g. recorded focused group discussions *versus* filled numerical data survey questionnaires), study designs (e.g. phenomenology *versus* cohort), analysis methods (e.g. descriptive phenomenological analysis *versus* software based statistical analyses)—their underpinning variation generally lies on the assumptions about the world, reality, science and knowledge [2,3]. Eventually these variations determine the choice of research approach among researchers. For a phenomena or research problem with little is known about and that needs a researcher's effort to explore, uncover, describe and understand; qualitative approach is better alternative than the quantitative one. Besides, qualitative research also serves as a springboard providing research questions and testable hypothesis for the later quantitative studies, and most importantly, it ‘humanizes’ the sense of enquiry [4]. Somewhere in between the two sides of quantitative-qualitative tradition fence of research, found on separate paradigms [5,6], mixed method emerged in response to 1970s and 1980s positivists versus constructivists “paradigm war” among the respective purists [7]. Although it is perceived to fill shortcomings of qualitative-quantitative dichotomous view in research, I believe discussing mixed method and its paradigms and assumptions is of less relevance here for now.

Except a handful of educational studies mainly published in non-positivist oriented social science and nursing journals, authors of medical researches fail to explicitly determine their paradigms, assumptions, methodologies, theoretical frameworks, etc. in their articles ultimately affecting the much anticipated ‘academic legitimacy’ [8]. One's research philosophical inclination or paradigm preference determines the whole process of the study as presented in subsequent sections below, and it also affects understanding of research findings by intended end users. An important feature of qualitative research, perhaps idiosyncratic, is the use of theories. Specific place where and the time when to use theories in qualitative approach has remained an area of scholarly debate. In a qualitative approach, theory can be used for multitude of purposes at different stages of the research processes – during the development of research question and justification of the rational of its methodology, as a lens of seeing at the target phenomena during the design and data collection, framing data analysis and result interpretation, and finally serves as triangulation tool of study findings during write up [9]. Hence, theory of development of medical expertise (TDME) used as a major theory for a purpose of framing result interpretation in the qualitative study that we conducted about OSCE experience. I believe the challenges and experiences during our qualitative study have what it takes to be learning for other researchers. Therefore, this article, as modified extension of methods section of a phenomenological study about exploring OSCE exam experience of clinical year-II medical students who were on the attachment of Obstetrics and Gynecology department and their educators, briefly discusses the research paradigm and the philosophical assumptions leading to specific qualitative approach and its entire processes in the following sections.

## 2 Philosophical assumptions and research paradigm

Philosophical positions towards— ‘*what’ and ‘how’ about of*— reality, knowledge and world generally determine the research paradigm and traditions. The basic philosophical concepts in this regard are ontology, epistemology and methodology. To begin with simpler concept, I cite Crotty stating methodology is the strategy, plan of action, process or design lying behind the choice and use of particular methods and linking the choice and use of the methods to the desired outcomes [10]. I believe the three concepts— i.e. ontology, epistemology and methodology— have non-linear iterative relations one affecting another and influence the way a researcher approaches the world. In addition to framing the methodology, explicit description of ontological and epistemological stances helps readers easily understand the research finding.

## 3 Ontological and epistemological assumptions

Ontology is an assumption we make about the kind and nature of the reality, what exists and the social world itself [11,12]. Simply put, it is “study of being” [10] and answers questions *‘what is there that can be known?’* and *‘what is the nature of reality?’* [13]. Based on Bryman's “Social ontology” social entities or realities are either objective entities which exist independently from social actors or social constructions in themselves built up from the perceptions, actions and interpretations of the individuals in society [14]. The latter assumption of Bryman fits to our enquery and serves as ontological foundation. Epistemology is an assumption we make about kind or nature of knowledge [11], and how we look at and make sense of the world [15]. Simply put, it is nature and form, way of acquisition and communication of knowledge. There are two dominant schools of thought as far as ontological and epistemological traditions towards reality and knowledge are concerned: Objectivism/Positivism and Interpretivisim/Constructivism. From positivist/objectivist perspective knowledge is viewed as hard, tangible, measurable, static and value free where a researcher distances self from research so as to merely observe, measure and test, etc., without any impact on the finding. From Interpritivist and constructivist perspective, on the other hand, knowledge is viewed as subjective, personal, unique and flexible where researcher is engaged with the subjects. Whereas the former is highly associated with quantitative research tradition, the latter is related to qualitative research traditions. Hence, ontological assumption affects our epistemological inclination which in turn influences research method and design. Interpritivist/constructivist tradition frames the methodological approaches of our study which is phenomenological qualitative design.

## 4 The qualitative approach: phenomenology

Based on the research question, the phenomena to be interpreted, understood, and generated as knowledge by the researcher in our enquiry was the experience and challenge of OSCE as perceived by students and educators. Phenomenological approach fits to the research question under investigation. The purpose of phenomenological research is to understand the essence of social phenomena from the perspective of those who perceived it [16]. Therefore, this approach enables us to understand the nature and meaning of students' and examiners' OSCE experience and perceived challenges in simulation center. The most common variations of phenomenological studies are descriptive (Husserlian) phenomenology and Interpretative Hermeneutic (Heideggerian) phenomenology [17]. Descriptive phenomenology is important approach in areas where there is little or no previous research evidences exist [18], which supports our choice in the enquiry. It is believed to have scientific rigor in that researcher puts aside his/her preconceptions in a method called bracketing. Descriptive phenomenology, as coined by Collaizi, has seven steps of analysis. However, Giorgi who had proposed step wise analysis approach of descriptive phenomenology before Collaizi's discovery of seven steps analysis approach, later criticized the necessity of returning research finding to participant for further verification at seventh step [19]. I agree with Collaizi's criticism of seven steps approach and decided to complete the analysis at sixth step. The assumptions and descriptions of modified Callaizi's descriptive phenomenological analysis will be briefly discussed in data analysis sub topic below:

### 4.1 Data collection method

In descriptive phenomenology, although the “rich first-person accounts of experience” like face to face interview is preferred, other methods such as written narratives, blogs, research diaries, online interviews, etc., can be employed as data collection tools [18]. On the other hand, Marshal suggested possibility of employing open-ended structured interview using questionnaire to explore topics ranging from cultural differences, first hand encounters, and the perceptions, meanings, and interpretations of participants [20]. I believe self-administered or self-report open ended structured interview would fit to our study design and explore factors affecting OSCE implementation and challenges as perceived by students and instructors. Therefore, in our study the sample of 49 C-II medical students and seven examiners from department of Ob-Gyn were interviewed using self-report written questionnaire. In this regard, Elliott and Timulak in a chapter of a book, *Descriptive and interpretative approach of qualitative research*, recommended that self-report questionnaire with open ended questions can be used for qualitative data collection, but the best practice in such case would be to keep participants' telephone or email contact in advance until the time of data analysis to seek any further elaboration on the points they responded [21]. Fortunately there were no responses for which we sought further elaboration and we didn't contact study participants even though we had their addresses. To minimize “memory decay, alterations or participant response errors” related to self-report interview in phenomenological study [22], questionnaire was distributed immediately after participants finished their OSCE exam. With modification in a way it suits to the phenomenological enquiry when supplemented with orientation—that their experience and perception is needed— before data collection, questionnaire was adapted from previous qualitative study [23]. The authors of the previous study explored students' perception through qualitative evaluation of their feedback using structured self-report interview. It has some implicit similarity—ontologically and epistemologically—with our enquiry although there is minor methodological difference. To give phenomenological sense to the adapted questionnaire, participants in our study were verbally informed in advance to express their feeling about OSCE experience and perceived challenges while answering the questions. Data was collected at the end of summative OSCE exam session using adapted tool consisting of the questions below (Table 1).

**Table 1:** Data collection tool in our previous study.

No.	Questions
	Did you have OSCE experience before? *If yes for Q no.1, how many times did you examined (with) OSCE?*
2	What do you think about the structure of OSCE?
3	Was the number of OSCE stations adequate*? (if Yes/No, Explain your reason*.
4	Was the time allocated in each station fair/time management adequate for OSCE stations? *(If Yes/No, Explain your reason).*
5	Did the OSCE stations sufficiently cover the major areas of your course or attachment (Ob-Gyn)? *(Explain your reason)*
6	What do you feel if there was written stations in addition to skill/procedure stations?
7	Was the OSCE format easy to follow? *(think of instructions, scenarios, materials, examiners, etc and explain)*.
8	What did you like about this OSCE exam?
9	What did you not like about this OSCE exam?
10	How the OSCE can be improved? *Any recommendation?*
11	Generally *(not specific to this OSCE),* what is challenging or difficult for you when you prepare for OSCE?

#### 4.1.1 Data analysis

**Fig. 1:**
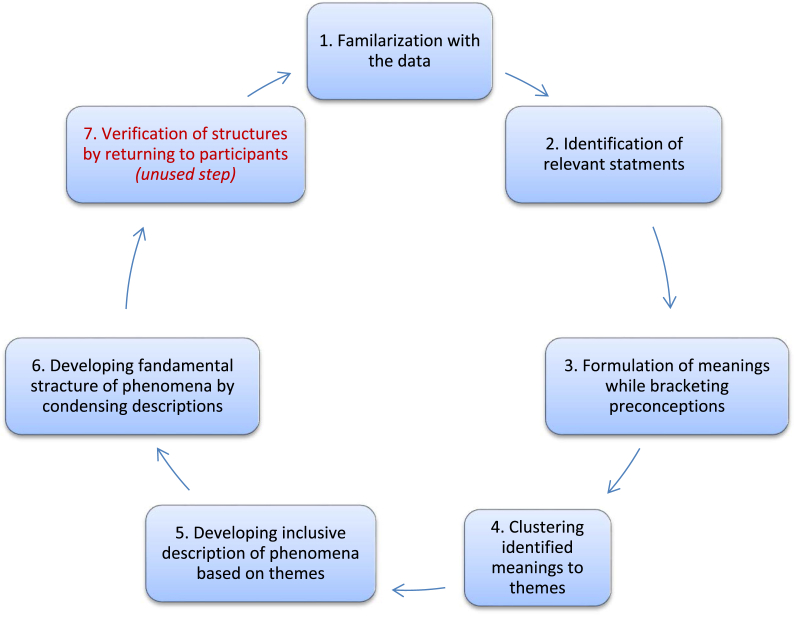
Collaizzi's seven steps descriptive phenomenological analysis.

In our study data analysis follows the Collaizzi's approach of descriptive phenomenological analysis. Basically, Collaizzi proposed seven steps where the first step is familiarizing oneself with the data and the final step being validating the finding by returning it to the study participants (Fig. 1). However, agreeing with Georgi's critics towards the seventh step of Collaizzi's method [19], I finalized analysis at sixth step. Perspective and attitude discrepancy among study participants and the phenomenology oriented researchers and also indefensible study result approval by same participant as ‘correct’ [18], besides difficulty to get all participants as concerned as they had been, were vindications for omission of the seventh step of Collaizzi's method in our study. Previous study by Obizoba, aimed to explore the strategies for mitigating the challenges of OSCE in baccalaureate nursing education program, was used as a benchmark for data analysis [24]. That study employed Collaizzi's seven steps descriptive phenomenological analysis although there is some discrepancy in data collection. Whereas Obizoba had utilized semi-structured interview along with observation as data collection method, structured interview was employed as sole data collection method in our study.

#### 4.1.2 Validity: reflexivity and bracketing (*Epoche*)

Validity can be enhanced anywhere throughout research process, most commonly during data collection and analysis. In the literature there are several strategies of increasing validity of qualitative study. This includes internal auditing, validation by research participants, triangulation with data of different sources, larger data to allow adequate saturation, and resonation with reader. Of all, internal auditing throughout the whole process, larger data and “reader resonation” were the strategies of validation employed in our study. Feedback from the advisors was used as important “reader resonation” strategy of validation. On the other hand, being an investigator of the study, I had experience of working with supervisory teams and examiners of OSCE for medical and health science students as a coordinator of clinical skills lab where students’ clinical competency assessed formatively and aware of some of challenges faculty members and students had been facing. This experience, which was consciously ignored throughout the study, served as just a base to understand and develop the research question.

Bracketing is the most important feature that differentiates Husserlian phenomenology from the Hermeneutic phenomenology. It demonstrates the validity of research process [25]. By doing so the researcher puts aside his/her preconceived knowledge, values, belief and experience while conducting the phenomenological analysis. There are four strategies of ensuring bracketing: Mental preparation, deciding the scope of literature review, planning data collection method immune to influences, and conducting analysis that enhance trustworthiness [26]. Regarding mental preparation during question development I defined my philosophical stand point and reassure myself that I would be open mindedly learn about the phenomena. Initial literature review was limited to just grasping enough understanding about the research question and suspending the exhaustive literature review for post analysis time. In contrast to the commonest phenomenological data collection method which is semi-structured interview, I used structured interview with predetermined open ended questions and bias due to posing leading questions wasn't a problem in our study. The importance of bracketing strategy during analysis is beyond just bracketing; instead it is attributed to the overall validity of the study. Descriptive phenomenological analysis by itself is more valid, scientifically speaking, than interpretative phenomenology. Moreover, applying the Collaizzi's descriptive analysis increases the overall trustworthiness particularly when the finding is returned to the participant at the seventh step of the analysis.

### 4.2 Strength and limitation from the study

Use of self-administered questionnaire for the structured interview minimized interviewer bias. Participants were interviewed immediately after OSCE exam experience and in so doing self-report related “memory decay, alterations or participant response errors” were minimized and considered as a strength of this phenomenological study.

The students' response rate (64.5%) was too low for qualitative survey but the sample size (49) was more than enough for qualitative study that it didn't affect the analysis of the result. The main reason for nonparticipation was post exam fatigue. Self-administered report questionnaire used in our study also has drawbacks or limitations that are expected in self-reported data. The following are the general limitations of self-reported data [27]:

⁃ Remembering or not remembering experiences or events that occurred at some point in the past **(selective memory)**
⁃ Recalling events that occurred at one time as if they occurred at another time **(telescoping)**
⁃ The act of attributing positive events and outcomes to one's own agency, but attributing negative events and outcomes to external forces **(attribution)**
⁃ The act of representing outcomes or embellishing events as more significant than is actually suggested from other data **(exaggeration)**

## 5 Conclusion

Research paradigm preference and philosophical inclination determine research approach, design, analysis and interpretation of findings. Positivist/objectivist researcher follows the quantitative approach and statistical and context-free “unbiased” analysis and interpretation. On the other hand interpritivist/constructivist researcher follows the qualitative approach with more flexible designs where the context and researcher's engagement in the process are considered as assets of the study. Despite one dominates the other in the arena of medical education research, we cannot certainly brand one approach as superior over another as long as both approaches serve their purposes— be it generalizability of result or deeper understanding of phenomenon—within their respective traditions. In one way or another, medical education researches from either traditions lead to one goal, which is improving the quality of education. Phenomenology, as a qualitative design, suits the inquiry of exploring the perception and experience of participant towards a phenomenon. Among the two variations of design, descriptive phenomenology is important where little or none is known about the research problem under investigation unlike interpretative phenomenology. The recommended data collection method in descriptive phenomenological design is in-depth interview with semi-structured questions, yet self-administered interview with structured open ended questions can also be used. In our phenomenological study we implemented descriptive phenomenological analysis with Collaizzi's seven steps to analyze data collected using structured open-ended questions of self-administered interview. To avoid selective memory, telescoping, attribution and exaggeration that are inherent to self-administered questionnaire, in-depth interviews and FGDs can successfully be used in similar phenomenological designs.

## Ethical approval

It doesn't require ethical approval.

## Sources of funding

None.

## Author contribution

The author contributed in all steps of study and manuscript preparation.

## Trial registry number

1. Name of the registry:
2. Unique Identifying number or registration ID:
3. Hyperlink to the registration (must be publicly accessible):

## Guarantor

Getu Ataro (Msc., MHPE., Assistant professor of clinical anesthesia).

## Consent

None.

## Provenance and peer review

Not commissioned, externally peer reviewed.

## Declaration of competing interest

None. The author didn't receive any financial or any other support from anyone.
